# *In vivo* Gene Therapy to the Liver and Nervous System: Promises and Challenges

**DOI:** 10.3389/fmed.2021.774618

**Published:** 2022-01-18

**Authors:** Alessio Cantore, Alessandro Fraldi, Vasco Meneghini, Angela Gritti

**Affiliations:** ^1^San Raffaele Telethon Institute for Gene Therapy, Istituto di Ricovero e Cura a Carattere Scientifico San Raffaele Scientific Institute, Milan, Italy; ^2^School of Medicine, Vita-Salute San Raffaele University, Milan, Italy; ^3^CEINGE Biotecnologie Avanzate, Naples, Italy; ^4^Department of Translational Medicine, University of Naples “Federico II”, Naples, Italy

**Keywords:** gene therapy, liver, central nervous system, gene editing, translational medicine

## Abstract

*In vivo* genetic engineering has recently shown remarkable potential as a novel effective treatment for an ever-growing number of diseases, as also witnessed by the recent marketing authorization of several *in vivo* gene therapy products. *In vivo* genetic engineering comprises both viral vector-mediated gene transfer and the more recently developed genome/epigenome editing strategies, as long as they are directly administered to patients. Here we first review the most advanced *in vivo* gene therapies that are commercially available or in clinical development. We then highlight the major challenges to be overcome to fully and broadly exploit *in vivo* gene therapies as novel medicines, discussing some of the approaches that are being taken to address them, with a focus on the nervous system and liver taken as paradigmatic examples.

## Introduction

Gene therapy (GT) has recently gained renewed interest and shown remarkable potential as a novel effective treatment for an ever-growing number of diseases, as also witnessed by the recent marketing authorization of several gene therapy products (1). *In vivo* genetic engineering, i.e., GT, involves the direct delivery of a GT medicinal product (GTMP) to patients either *in situ* in anatomically defined locations or systemically to reach organs or tissues such as central and peripheral nervous system (CNS, PNS), liver, muscles, and lungs. Emerging technologies for targeted gene editing are complementing the scope of conventional gene transfer, opening the way to precise gene correction that allows to silence, activate, or rewrite loci of interests in the genome. The GTMP may comprise a virus-derived or non-viral vehicle bearing a transgene expression cassette (gene transfer) or engineered site-specific nucleases or genetic/epigenetic modifiers with or without an exogenous DNA to be introduced into the host cells' genome (gene editing) (2–4). Short interfering RNAs (siRNAs) will not be considered here as GTMPs.

*In vivo* genetic engineering aims at genetically modifying somatic cells to: (i) treat genetic diseases, by adding functional genes (gene addition) or replacing dysfunctional ones (gene replacement), correcting or disrupting mutated disease-causing genes (gene subtraction) through pre-natal, post-natal or adult intervention; (ii) promote endogenous regeneration by delivering factors for tissue protection/engineering; (iii) tackle cancer by direct/indirect tumor cell elimination, including the use of oncolytic vectors (this will not be discussed here).

The most widely used delivery system for *in vivo* GT among viral vectors are adeno-associated viral (AAV) vectors (5). Lentiviral vectors (LV) are so far mostly used for *ex vivo* GT approaches, i.e., genetic engineering of cells *in vitro* and infusion of the modified cells back to patients, with only few examples related to *in vivo* delivery at the pre-clinical or early clinical stage (6, 7). Lipid nanoparticles (LNP) or chemical conjugates are used for small RNA delivery (8). Non-viral mediated delivery of genome editing components is generally at an earlier stage of development. The vast majority of current clinical trials rely on gene addition, only a few of them are based on gene editing strategies.

The availability of programmable nucleases, such as zinc-finger nucleases (ZFN), transcription activator-like effector nucleases (TALEN) and, more recently, clustered regularly interspaced short palindromic repeat (CRISPR)–Cas-associated nucleases, has greatly expedited the progress of gene editing from concept to clinical practice (4, 9). Engineering of the Cas9 bacterial adaptive immunity response against phages allowed for the development of methods to generate sequence-specific modifications based on a single-guide RNA complementary to the target genomic sequence. In the last decade, CRISPR/Cas9 systems have been applied to genome and epigenome editing in order to disrupt genes, correct mutations, and silence disease-associated factors in different genetic and sporadic conditions. Genome editing has been predominantly performed *ex vivo*, however a few examples of *in vivo* gene editing exist in early-stage clinical trials.

**Figure 1:**
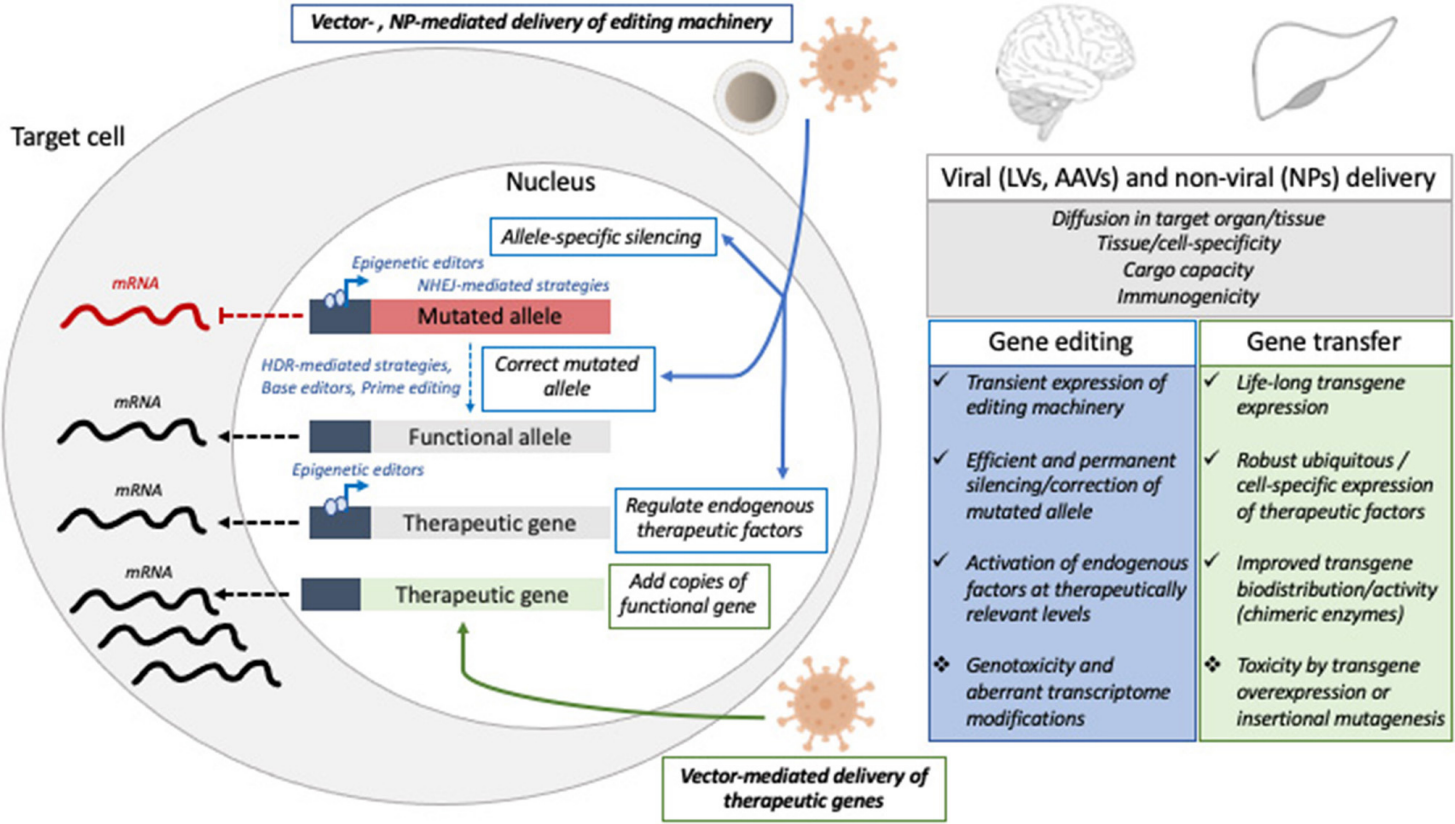
Schematic representation of gene editing and gene transfer approaches tested in pre-clinical and clinical settings to treat liver or CNS disorders, with a list of the major hurdles and challenges that might be addressed to improve the efficacy and safety of *in vivo* GTMP. NP, nanoparticles; LV, lentiviral vectors; AAV, adeno-associated viral vectors; NHEJ, non-homologous end joining; HDR, homology-directed repair.

Here we highlight the major hurdles currently limiting the full potential of *in vivo* genetic engineering (Figure 1) and review some possible solutions, with a focus on CNS and liver taken as paradigmatic examples.

## Commercial and Clinical Stage Products

### *In vivo* GT to the Nervous System

Currently, there are 3 commercial *in vivo* GT products and many more in clinical development (Table 1) (10). AAV vector-mediated gene replacement of a functional enzyme of the retinal pigment epithelium, or the regulatory protein survival of motor neuron is at the bases of Luxturna and Zolgensma, indicated for an inherited form of retinal blindness (Leber congenital amaurosis, LCA) or the genetic neurodegenerative disease spinal muscular atrophy, respectively (11, 12). Luxturna is administered *in situ* in the subretinal space, while Zolgensma is delivered systemically. In both cases, long-lasting therapeutic benefit has been shown, with remarkable recovery of vision and motor functions, respectively. Imlygic is an oncolytic vector indicated for melanoma (13).

**Table 1:** Commercial *in vivo* gene therapy products.

**Name**	**Indication**	**Vehicle**	**Mechanism**
Luxturna	Leber congenital amaurosis	AAV vector	Gene replacement
Zolgensma	Spinal muscular atrophy	AAV vector	Gene replacement
Imlygic	Melanoma	Herpes simplex type 1 virus-derived vector	Oncolytic vector Gene addition

Encouraging results have also been shown for Duchenne muscular dystrophy by systemic delivery of AAV vectors expressing short forms of dystrophin in early-stage clinical trials (14). Systemic, intrathecal, and intraparenchymal administration of AAV vectors is under early clinical testing for several neurodegenerative diseases, both genetic early-onset (mucopolysaccharidoses (MPS), globoid leukodystrophy (GLD), Fabry disease, Canavan disease) and non-genetic adult-onset diseases [e.g., Parkinson disease (PD), Alzheimer Disease (AD)] (15). Clinical trials involving LV as delivery systems for *in vivo* GT are currently limited to PD, which benefits from intrastriatal injections of a LV coding for three genes essential for dopamine synthesis (16).

EDIT-101 is a gene-editing drug to treat LCA10 with Centrosomal Protein 290 (CEP290)-Related Retinal Degeneration (17). The approach is based on AAV-mediated single-dose subretinal delivery of a CRISPR-Cas9 system designed to excise the intronic IVS26 mutation in the photoreceptor CEP290 gene that causes abnormal splicing and termination of translation due to introduced cryptic exon. EDIT-101 recently entered clinical testing and enrolled 18 people with LCA10 (NCT03872479). To date, no study report has been published.

### *In vivo* GT to the Liver

Systemic administration of AAV vectors expressing coagulation factor VIII or IX transgene in hepatocytes is in advanced phase of clinical testing as a treatment for the inherited coagulation disorder hemophilia and showed multi-year reconstitution of therapeutic amounts of the clotting factors, even though a decreasing trend in factor VIII activity has been reported (18–20). A similar strategy is under evaluation for some inherited liver metabolic diseases (such as familial hypercholesterolemia, hyperbilirubinemia, glycogen storage disease type-Ia, ornithine transcarbamilase deficiency) in earlier phase clinical trials (21).

NTLA-2001 is an *in vivo* gene-editing therapeutic that is designed to treat transtyrhetin (TTR)-related hereditary amyloidosis. Systemic administration of LNP delivering CRISPR/Cas9 RNA to the liver resulted in efficient disruption of TTR gene and subsequent reduction of the toxic misfolded TTR amyloid in 6 affected patients (22).

Despite these successes, several challenges remain to be addressed related to efficacy, safety and immunogenicity of *in vivo* GTMP, as well as manufacturing, regulatory aspects and sustainability, the latter not being the focus of this Mini Review. Below, we highlight the major challenges and elaborate on possible solutions to address some of them.

## General Challenges Related to Efficacy and Safety of *in vivo* Genetic Engineering

The efficiency of the genetic engineering (viral gene transfer or genome editing), i.e., the actual quantity of genetically modified cells/genomic loci, may be limiting the efficacy of the procedure depending on the desired therapeutic effect. Tissue/cell-type specificity may be desirable or necessary according to different applications, yet hard to achieve. While the tropism of viral vectors can be controlled to a certain extent, it is currently more difficult to obtain specific targeting by non-viral delivery systems (23, 24). On the other hand, cargo capacity may be more limited for viral than non-viral mediated approaches. Despite a plethora of engineered transcriptional and post-transcriptional control elements available, the strength, cell-type specificity, physiological regulation, duration of transgene expression may all be difficult to control and switching expression on and off at will is yet to be achieved in the clinics (25, 26). For genome editing, efficient but transient expression of the editing machinery should be achieved. For non-monogenic diseases, the target genes to manipulate need to be defined. Ensuring the multi-year, ideally life-long durability of the therapeutic genetic modification is crucial in the context of genetic diseases and needs to rely on transgene integration or stability of the genomic edit in proliferating cells and/or in long-lived target cells; alternatively, safe re-administration of the GTMP has to be ensured (27–29).

Concerning the safety of the *in vivo* genetic engineering, the following risks need to be taken into consideration, carefully evaluated, and reduced to the minimum possible during the research and development phases: the acute responses to the delivery vehicles (30, 31), toxicities due to expression/overexpression of the transgene or other components of the GTMP, possible long-term adverse effects due to genomic insertions of vectors or other components of the GTMP (32), genotoxicity associated with off-target events, large deletion at the on-target loci, chromosomal rearrangements, and aberrant modifications of the transcriptome (33–36). Moreover, the effects of the GTMP on target cells' biology and functionality should be properly determined. Finally, the innate and adaptive immune responses to the delivery vehicle(s), the transgene product(s), including editing machineries of bacterial origin, and other components of the GTMP need to be assessed to avoid detrimental impacts on both the efficacy and safety of the procedure (37, 38).

## Modifying the Transgene to Improve the Therapeutic Potential of *in vivo* GT

One of the main challenges in the clinical translation of *in vivo* GT is the difficulty in achieving and maintaining therapeutic amounts of the corrective gene in targeted tissues, avoiding the use of high dosage and/or repeated administration of the gene delivery vehicle (that, in most cases, is virus-derived), which is not only potentially toxic but also costly. Intra-vascular administration of GTMPs has been extensively tested in preclinical studies and is being exploited in clinical trials to treat the CNS as alternative approach to direct administration (*via* either intraparenchymal or intra cerebrospinal fluid injections), which in principle require lower amount of GMTPs but may represent an invasive approach. However, intra-vascular administration of GTMPs showed limited or no effectiveness on CNS pathology due to the impermeability of the blood-brain barrier (BBB) to large molecules (39). Therefore, this delivery route may require high doses of GTMPs, which may strongly reduce its clinical suitability. A possible strategy to overcome all these limitations is enhancing the therapeutic potential of the GTMP by modifying the expression cassette. Here, we give some examples on how this strategy can be applied to the treatment of inherited diseases due to enzymatic deficiency.

A way to modify the transgene expression cassette to enhance its therapeutic potential is adding specific peptides to generate chimeric enzymes with acquired capabilities. Lysosomal storage diseases (LSDs) are inherited metabolic conditions mostly caused by defective lysosomal hydrolases and often showing CNS involvement (40). The addition of heterologous signal peptides to soluble lysosomal enzymes has been showed to increase the secretion efficiency, thus improving enzyme bioavailability and tissue targeting upon *in vivo* GT in different models of LSDs, including MPS, GLD and Pompe diseases (41–44). In the case of Pompe disease, the liver directed administration of AAV encoding engineered secretable GAA (acid α-glucosidase) transgene in both mouse and non-human primate (NHP) animal models demonstrated improved efficacy associated to a clear dose advantage and reduced toxicity when compared to the native version of the GAA transgene. This approach is currently under clinical testing (NCT04093349). Furthermore, the fusion of the lysosomal hydrolase with specific protein domains capable to bind BBB receptors has been shown to allow active BBB crossing upon liver GT in preclinical LSD models. In these studies, the liver is converted into a factory for the engineered enzyme, which can cross the BBB and target the CNS upon secretion in the bloodstream (41, 45, 46). Interestingly, enzyme replacement therapy approaches based on the delivery of recombinant chimeric lysosomal enzyme fused to different BBB binding domains (BD) are under clinical evaluation for different MPS, thus supporting the potential clinical translation of GT protocols based on the viral mediated delivery of BBB-BD-modified enzymes.

An alternative way to enhance the therapeutic potential of the transgene is to use gain-of-function (GOF) mutants of the enzyme with increased activity and/or stability. Such “hyper functional” enzymes may be employed in *in vivo* gene transfer (as well as in enzyme replacement approaches) to produce a beneficial effect in targeted tissues at much lower doses and more efficiently compared to the respective WT enzymes. Naturally occurring GOF variants have already been used to treat liver diseases caused by inherited enzymatic defects. AAV vectors encoding a hyper-functional factor IX (FIX-Padua, R338L) has been explored for the treatment of hemophilia B. In dogs and mouse models of disease the use of such variant resulted in beneficial therapeutic effect and, at same time, allowed reducing the AAV vector dosage and, therefore, the risk of cellular immune response to vector capsid, which is one of the main complications of AAV GT for hemophilia B (18, 47, 48). In the case of lipoprotein lipase (LPL) deficiency (LPLD), an orphan disease associated with chylomicronemia, severe hypertriglyceridemia, metabolic complications, the use of AAV vectors encoding a GOF gene variant of LPL (S447X), showed efficacy in LPLD patients avoiding safety concerns related to immune response to AAV-capsid proteins (49). The possibility of generating GOF versions of enzymes “*ad hoc*” may greatly extend the possibility to apply safe GT protocols for the treatment of other metabolic diseases.

## Ensuring Durability of Liver Gene Therapy for Monogenic Diseases

Gene therapy for monogenic diseases promises to be a once-in-a-lifetime treatment that could be delivered at young age and last life-long. The clinical success obtained by AAV vector-based liver GT in adults with hemophilia has raised the expectation to extend enrollment to pediatric patients to maximize the potential benefits for the patients and to broaden the indications to diseases that are more severe or lethal in childhood, such as inherited diseased of liver metabolism. Because AAV vectors do not actively integrate into the host cell genome, they are progressively diluted upon cell division in liver growth, thus challenging their use in pediatric patients. To address this issue, AAV re-dosing, integrating vectors and genome editing and are being explored.

The anti-vector immune response induced after the first administration indeed currently limits the efficiency of a second administration, thus efforts are underway to counteract the anti-AAV immune responses and allow effective re-administrations (50–52). LV integrate into the target cell chromatin and are maintained as cells divide, thus being suited for stable and potentially life-long transgene expression even following a single administration to newborn individuals. Systemic i.v. (intravenous) administration of LV has been shown to allow efficient and long-term gene transfer to the liver and achieve phenotypic correction of hemophilia in mice and dogs (53, 54). Allo-antigen free and phagocytosis-shielded LV have been generated, by high-level surface display of the phagocytosis inhibitor human CD47 (CD47hi) (55, 56). Following i.v. administration to NHP, these CD47hi LV provided amounts of circulating human coagulation factor transgene that would be therapeutic for hemophilia, the disease caused by the deficiency of one of these factors, without evidence of acute toxicity or genotoxicity. These LV are under development for clinical evaluation in hemophilia (57).

Site-specific integration of a corrective DNA in the genome remains an attractive therapeutic strategy for genetic diseases and represents an area of active investigation. The first report of successful *in vivo* genome editing in the liver in mice by ZFN dates back in 2011 by the K. High group, in collaboration with Sangamo Therapeutics (58, 59), an approach which has been later brought to early clinical testing in the context of Hunter's syndrome (60). The trial has been then closed and the results have not been published yet. In 2015, Barzel et al., reported a nuclease-free genome editing approach in the mouse liver, based on the spontaneous tendency of AAV vectors to integrate on a homology-dependent basis (61). This approach is being brought to early clinical testing in the context of the metabolic disease methyl malonic acidemia (https://investor.logicbio.com/news-releases/news-release-details/logicbio-therapeutics-announces-first-patient-dosed). Instead, Yin et al. reported in 2014 the first report of hepatocyte gene editing mediated by CRISPR/Cas9 for hereditary tyrosinemia type-I in mice (62). More recently, the advent of base editors has opened the possibility to perform single-base substitutions for therapeutic purposes (63). The availability of the highly efficient and transient LNP-based mRNA delivery system recently enabled nuclease-mediated or base-editor mediated genome editing in the liver of NHP and even humans (64, 65). Recently the results of the first clinical trial exploiting genome editing directed to the liver have been reported. These results showed high efficiency of gene disruption and evidence of therapeutic efficacy for the autosomal dominant disease TTR amyloidosis (22). The most advanced genome editing therapies remain so far mostly confined to gene subtraction approaches, however these encouraging results will fuel further progress toward more challenging gene correction approaches. Vector re-administration, integrating gene replacement and editing strategies have all advantages and disadvantages, thus extensive pre-clinical evaluations and risk/benefit assessments need to be conducted on an indication-per-indication basis.

## Enhancing the Distribution and Cellular Selectivity of GTMPs to Improve *in vivo* CNS GT

The route of administration, the vector tropism, and the regulatory elements driving transgene expression are key determinants in defining the efficacy and safety of *in vivo* GT to treat CNS disorders.

In focal neurodegenerative disorders, intraparenchymal administration in the affected regions is well-tolerated and ensures a local distribution of the GTMP with low vector doses, thus reducing off-target effects in peripheral organs and immunogenicity (15). Convention enhanced delivery has been exploited to further increase the diffusion of the vector in the brain parenchyma by generating a pressure gradient in the infusion catheter leading to expansion of the extracellular space (66). The overall safety of intraparenchymal administration of AAV vectors has been shown in pediatric and adult patients affected by genetic (e.g., Canavan disease, Metachromatic Leukodystrophy, Batten's disease) and non-genetic (e.g., PD, AD) CNS disorders (66, 67). LV are alternative GT vehicles ensuring stable and robust expression of therapeutic transgenes in disease-bearing cells with negligible immune reactivity (68–72). The higher LV cargo capacity can be exploited to deliver multiple genes regulating metabolic processes that are hampered in genetic (i.e., GM2 gangliosidosis) (72) and sporadic (i.e., PD) diseases (70, 71). Indeed, the 8-year follow-up on ProSavin, a LV delivering key enzymes of the dopamine biosynthetic pathway, documented an improvement of the “off state” time in 8/15 treated PD patients, with GTMP-unrelated mild-to-moderate adverse events (16). Intrathecal or systemic administration can ensure a widespread biodistribution of viral vectors resulting in effective targeting of the spinal cord and in the rostro-caudal coverage of different brain regions (73). These approaches are better suited for the treatment of multifocal/diffuse neurodegenerative diseases (66, 67), including GM2 gangliosidosis (74). Still, they require higher vector doses and enhance targeting of off-target tissues, dorsal root ganglion pathology, and immune response against the GTMP (75, 76).

The selective delivery of GTMPs to the target cell populations/cell subtypes is necessary to improve both the efficacy and safety of GT. The efficiency of AAV vectors and LV in targeting different neuronal populations has been proven in rodents and NHP (15). The higher tropism of LVs for oligodendrocytes (69, 77–79), astrocytes (80) and microglia (81, 82) defines these vectors as a good candidate for gene transfer in glial populations. Recently, AAV hybrid serotypes and AAV variants generated by directed evolution or structural mutagenesis have been selected for their enhanced transduction efficiency in macroglia cells (83–85). In particular, systemic administration of the AAV9 variant AAV-F showed high proficiency for astrocyte transduction and a CNS distribution similar to the BBB-crossing AAV9.PHP.B variant (86), suggesting their potential use for less invasive targeting of cells involved in neuroinflammation processes.

The cell specificity of GTMPs could be enhanced by the inclusion of lineage-specific regulatory elements in the transgenic constructs. The size of cell-type specific promoters has been shortened to fit AAV cargo capacity and tested in pre-clinical models, resulting in upstream regulatory elements able to enhance and/or restrict transgene expression in neurons (e.g., NSE, CaMKII and Syn1 promoters) (87), astrocytes (e.g., gfaABC (1)D promoter) (84), oligodendrocytes (e.g., *Mag* promoter) (88), or microglia and brain-infiltrating macrophages (e.g., F4/80 and CD68) (89, 90). De-targeting strategies based on endogenous microRNAs selectively expressed in off-target cell populations could further increase cell-specific transgene expression (81, 82), decrease the targeting of off-target cells/tissues (91, 92), and mitigate immune responses (93). The multiplexing of different microRNA de-targeting strategies favors the refinement of the post-translational regulation of transgene expression.

Nanoparticles (NPs) delivering large-size Cas9 nucleases, genome or epigenome modifiers are the ideal GTMP vehicle to ensure effective on-target editing by transient and safer expression of the editing machinery. Intraparenchymal injection of CRISPR/Cas9-loaded NPs have been tested in animal models to treat focal neurodegenerative disorders, such as Fragile X syndrome (94) and AD (95). The limited distribution and rapid clearance of NPs hamper their application in multifocal neurodegenerative diseases, for which multiple site administration or NP functionalization to increase cell-specific uptake and the BBB crossing are required to ensure CNS distribution upon systemic injection (96). Future *in vivo* validation of NP platforms to deliver GTMPs in the brain of large animals is a crucial step in the long path toward their clinical applications.

## Conclusion

*In vivo* genetic engineering has experienced considerable progress in the last decade and a few landmark studies have convincingly shown that somatic genetic modification for therapeutic purposes can be safely achieved in humans. These new advanced therapies remain highly complex, only partially understood, difficult and costly to develop. Yet, they hold tremendous therapeutic potential and promise to revolutionize medicine. We have highlighted some of the many challenges that still need to be addressed and some avenues that are being explored for broader exploitation and effective introduction of these therapies into clinical practice. To achieve this goal, technical advances need to be accompanied by a continuous dialogue and cooperation between academia, biotechnology and pharmaceutical companies, regulators, policy makers and a civil society with high education and trust in science.

## Author Contributions

AC, AF, VM, and AG: wrote the manuscript. All authors contributed to the article and approved the submitted version.

## Funding

This work was supported by Italian Ministry of Health (GR-2019-12368956) to AC; Cure Sanfilippo Foundation and Sanfilippo Children's Foundation (Joint grant 2021-2023), Fondazione Telethon (Tigem Core grant 2016-2020) to AF; Fondazione Telethon (Tiget Core Grant 2016-2021, grant D2), European Leukodystrophy Association (ELA 2019-015I2), European Joint Program on Rare Diseases (EJPRD; project NG4Leuko) to AG; EU Marie Curie fellowship (895111 ASTRO-EDITING), Italian Ministry of Health (GR-2019-12368930), ELA (ELA 2020-010I2) to VM.

## Conflict of Interest

The authors declare that the research was conducted in the absence of any commercial or financial relationships that could be construed as a potential conflict of interest.

## Publisher's Note

All claims expressed in this article are solely those of the authors and do not necessarily represent those of their affiliated organizations, or those of the publisher, the editors and the reviewers. Any product that may be evaluated in this article, or claim that may be made by its manufacturer, is not guaranteed or endorsed by the publisher.

## Abbreviations

AAV: adeno-associated viral
AD: Alzheimer Disease
BBB: blood-brain barrier
CNS: central nervous system
CRISPR: clustered regularly interspaced short palindromic repeat
GAA: acid α-glucosidase a pag. 6
GLD: globoid leukodystrophy
GOF: gain-of-function
GT: gene therapy
GTMP: gene therapy medicinal product
i.v.: intravenous
LCA: Leber congenital amaurosis
LNP: lipid nanoparticles
LPL: lipoprotein lipase
LPLD: LPL deficiency
LSD: lysosomal storage diseases
LV: lentiviral vectors
MPS: mucopolysaccharidoses
NHP: non-human primate
PD: Parkinson's disease
PNS: peripheral nervous system
TALEN: transcription activator-like effector nucleases
TTR: transthyretin
ZFN: zinc-finger nucleases.
